# Bulge, Bubble, and Y: How an RNA Exonuclease Repairs DNA, in Detail

**DOI:** 10.1371/journal.pbio.1001804

**Published:** 2014-03-04

**Authors:** Richard Robinson

**Affiliations:** Freelance Science Writer, Sherborn, Massachusetts, United States of America

For a cell, it's not so much death or taxes, but damage to its DNA, that is inevitable. Chemicals, radiation, and ultraviolet light all take their toll, disrupting the double helix and leaving dangerous chaos in their wake. The genome in virtually every cell in every organism suffers such damage multiple times over its lifespan. Left unrepaired, such disruptions would quickly leave the cell either unable to survive, or perhaps unable to check its own growth. Many tumors get their start when a damaged gene is unsuccessfully repaired.

**Figure:**
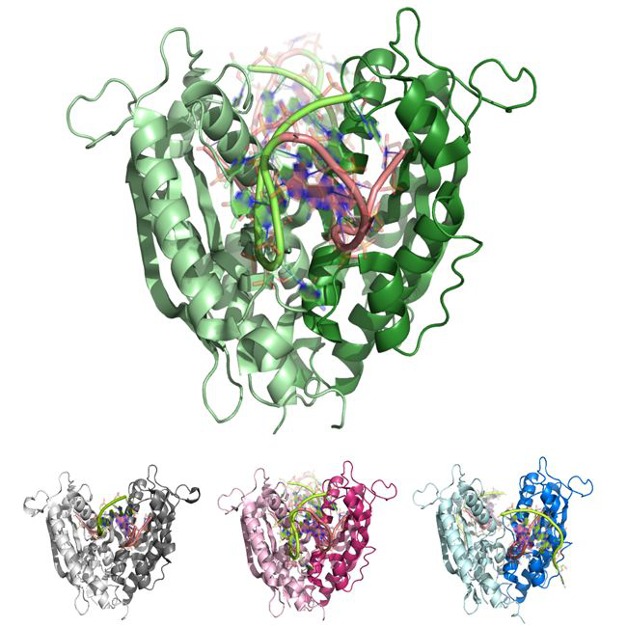
The exonuclease RNase T is a versatile protein that can recognize and bind varied DNA structures and cleave DNA in different ways.

Fortunately, cells have an extensive set of repair enzymes that together cut, cleave, replace, and stitch back together damaged DNA. In this issue of *PLOS Biology*, Yu-Yuan Hsaio, Hanna Yuan, and colleagues significantly expand the known repertoire of one repair enzyme, and show in detail how it helps fix three different types of damage.

RNase T cleaves RNA, but is also known to act on DNA, at least in vitro. It is an exonuclease, meaning it cuts nucleotides off the end of a DNA strand (an endonuclease would cut in the middle of a strand). The end of a properly paired helix that is cleaved cleanly across, with no extra unpaired nucleotides on either side, is called a “blunt end duplex.” RNase T cannot act on such structures. The anti-parallel structure of DNA means that the two free ends of the helix are chemically distinct; one nucleotide has its so-called 5′ end exposed, the other its 3′ end. RNase T cleaves unpaired 3′ ends, one nucleotide at a time.

Working in bacteria, the authors began by showing that RNase T was essential for DNA repair, and responded to damage induced by multiple sources, including hydrogen peroxide and ultraviolet light. They found that the enzyme aided in repairing three distinct types of damage-related structures, called bulges, bubbles, and Y ends. In a bulge, base pairing is preserved at the very end of the helix, as it is in a blunt-end duplex, but there is a mismatch in the penultimate bases, causing the two sides of the helix to bulge out behind the blunt end. If a mismatch occurs further within the helix, it forms a bubble instead. If there is no base pairing at the end, the two ends float free of each other, forming a “Y.”

To determine how the enzyme was able to cleave the blunt end of a bulge, but not the blunt end of a properly formed duplex, the authors turned to X-ray crystallography of the enzyme with DNA substrate in place. They found that a critical role was played by a phenylalanine near the active site. When binding a duplex, that phenylalanine formed an association with the base on the 5′ side of the helix, stopping further cleavage. But when a bulge was present, the phenylalanine group's attraction for neighboring bases on the 5′ side instead promoted cleavage of the bulging nucleotides on the opposite side.

When acting on single-strand RNA or DNA, RNase T is restricted in a sequence-specific way, slowing when confronted with a C nucleotide, and stopping in its track when two C's occur together. Curiously, those restrictions were absent when it digested double-stranded DNA. Here, the key role was played by the helix itself—when a terminal C was base-paired with G, C was unable to interact with a glutamic acid, the source of slowing.

The authors also discovered an unexpected relationship between the enzyme and Y-structured DNA. The structure of the helix-enzyme complex suggests that if the free 5′ end of the Y were oriented as it is when a bulge is processed, the 5′ end would likely interact with the phenylalanine and interfere with the ability of the 3′ end to proceed into the active site. Instead, X-ray crystallography revealed that the helix is rotated 180° along its long axis, keeping the 3′ end at the active site, but transferring the 5′ end to a distal site on the enzyme where it can increase, rather than reduce, affinity of the DNA for the enzyme.

Since the enzyme is an exonuclease, it is unable to digest DNA at bubbles. By examining repair under a variety of conditions, the authors showed that RNase T likely acts downstream from the endonuclease Endo V, which first cuts near the bubble to expose a free end.

The mechanistic insights provided by this study go far beyond this single enzyme, since there are thousands of distinct enzymes that have 3′ exonuclease activity. While some of the details will no doubt vary, it is likely that many of these enzymes use similar mechanisms.

**Hsiao Y-Y, Fang W-H, Lee C-C, Chen Y-P, Yuan HS (2014) Structural Insights Into DNA Repair by RNase T—An Exonuclease Processing 3′ End of Structured DNA in Repair Pathways** doi:10.1371/journal.pbio.1001803.

